# AhR Activation Leads to Alterations in the Gut Microbiome with Consequent Effect on Induction of Myeloid Derived Suppressor Cells in a CXCR2-Dependent Manner

**DOI:** 10.3390/ijms21249613

**Published:** 2020-12-17

**Authors:** Wurood Hantoosh Neamah, Philip Brandon Busbee, Hasan Alghetaa, Osama A. Abdulla, Mitzi Nagarkatti, Prakash Nagarkatti

**Affiliations:** Department of Pathology, Microbiology & Immunology, University of South Carolina School of Medicine, Columbia, SC 29209, USA; wurood814@gmail.com (W.H.N.); brandon.busbee@uscmed.sc.edu (P.B.B.); Hasan.Alghetaa@uscmed.sc.edu (H.A.); Osama.Abdulla@uscmed.sc.edu (O.A.A.); mitzi.nagarkatti@uscmed.sc.edu (M.N.)

**Keywords:** 2,3,7,8-tetrachlorodibenzo-p-dioxin, aryl hydrocarbon receptor, microbiome, myeloid-derived suppressor cell, CXCR2

## Abstract

Aryl hydrocarbon receptor (AhR) is a ligand-activated transcription factor and 2,3,7,8-tetrachlorodibenzo-p-dioxin (TCDD) is a potent ligand for AhR and a known carcinogen. While AhR activation by TCDD leads to significant immunosuppression, how this translates into carcinogenic signal is unclear. Recently, we demonstrated that activation of AhR by TCDD in naïve C57BL6 mice leads to massive induction of myeloid derived-suppressor cells (MDSCs). In the current study, we investigated the role of the gut microbiota in TCDD-mediated MDSC induction. TCDD caused significant alterations in the gut microbiome, such as increases in *Prevotella* and *Lactobacillus*, while decreasing *Sutterella* and *Bacteroides*. Fecal transplants from TCDD-treated donor mice into antibiotic-treated mice induced MDSCs and increased regulatory T-cells (Tregs). Injecting TCDD directly into antibiotic-treated mice also induced MDSCs, although to a lesser extent. These data suggested that TCDD-induced dysbiosis plays a critical role in MDSC induction. Interestingly, treatment with TCDD led to induction of MDSCs in the colon and undetectable levels of cysteine. MDSCs suppressed T cell proliferation while reconstitution with cysteine restored this response. Lastly, blocking CXC chemokine receptor 2 (CXCR2) impeded TCDD-mediated MDSC induction. Our data demonstrate that AhR activation by TCDD triggers dysbiosis which, in turn, regulates, at least in part, induction of MDSCs.

## 1. Introduction

2,3,7,8-Tetrachlorodibenzo-p-dioxin (TCDD) is an environmental pollutant generated during the manufacture of herbicides or burning organic materials such as waste incineration, fossil fuels, and wood combustion [1]. Ingestion of contaminated food is one of the most common exposures to TCDD in humans [2]. Following TCDD exposure, immune cells such as regulatory T cells (Tregs) are expanded and effector T-cells are suppressed [3], in addition to depletion of macrophages and dendritic cells in the jejunum [4,5]. A single dose of oral TCDD administration decreased immunoglobulin (Ig) A secretion in the gut by impairing B-cell function [6]. The gut is lined with a single layer of epithelial cells connected by tight junction proteins and is interspersed with mucus-secreting goblet cells and paneth cells, which release antimicrobial peptides [7]. Interestingly, mice treated orally with TCDD developed tolerance to ovalbumin (OVA) and showed suppression in the humoral immune response in the epithelial cells of the lumen, as well as serum and fecal samples [8]. However, in these same studies, alterations of other immune cells (e.g., CD4^+^, CD8^+^, CD19^+^, and CD103^+^MHCII^+^CD11c^+^) by TCDD occurred only in the gut-specific draining lymph node (MLN).

The diversity of microbes within a given host can be defined by the number, abundance, and distribution of distinct types of organisms such as bacteria, archaea, protists, fungi, and viruses. The interaction and activity of these microbes has been linked to the homeostasis of the immunologic, hormonal, and metabolic processes of the host. Microbial dysbiosis and alterations in the microbiome with negative effects on the host occur due to a wide range of causes such as diseases, environmental contamination, diet, and stress [9,10,11]. The gastrointestinal tract is the largest habitat of the microbiota, and emerging studies have shown that TCDD exposure leads to its rapid absorption into the gastrointestinal tract, which can cause shifts in gut microbiome commensals [12,13]. Nonetheless, whether there is a link between immunological changes induced by TCDD and the gut microbiota has not been previously studied.

Most of the activity of TCDD is mediated through the aryl hydrocarbon (AhR), a cytosolic-bound receptor expressed in a variety of immune cells including T-cells, monocytes, granulocytes, myeloid-derived suppressor cells (MDSCs), and mast cells. AhR activation by TCDD leads to alterations in the immune system involving several mechanisms such as disruption of the Treg/Th17 balance, suppression of the cytotoxic T-cell response, impairment of antibody production by B-cells in a T-cell dependent manner, a decrease in IL-6 and tumor necrosis factor (TNF) production by macrophages, and induction of apoptosis in activated T-cells [14,15,16,17,18,19]. TCDD is also well characterized for its ability to induce Tregs [14,20]. Recently, we observed that TCDD administration leads to a massive induction of MDSCs and MDSC subsets (granulocytic and monocytic) that are highly immunosuppressive and regulated by CXC chemokine receptor 2 (CXCR2) [21]. Thus, in the current study, we investigated if the microbiota of the host plays any role in the induction of MDSCs by TCDD.

Using 16S rRNA sequencing of the gut microbiome, we noted that TCDD exposure resulted in alterations of the gut microbiome and metabolome, such as a reduction in cysteine metabolism. Importantly, using fecal material transfer (FMT) experiments, we found that MDSC induction by TCDD was dependent, at least in part, on the gut microbiota.

## 2. Results

### 2.1. TCDD Exposure Alters the Gut Microbiome Composition and Short Chain Fatty Acid Production

**Figure 1:**
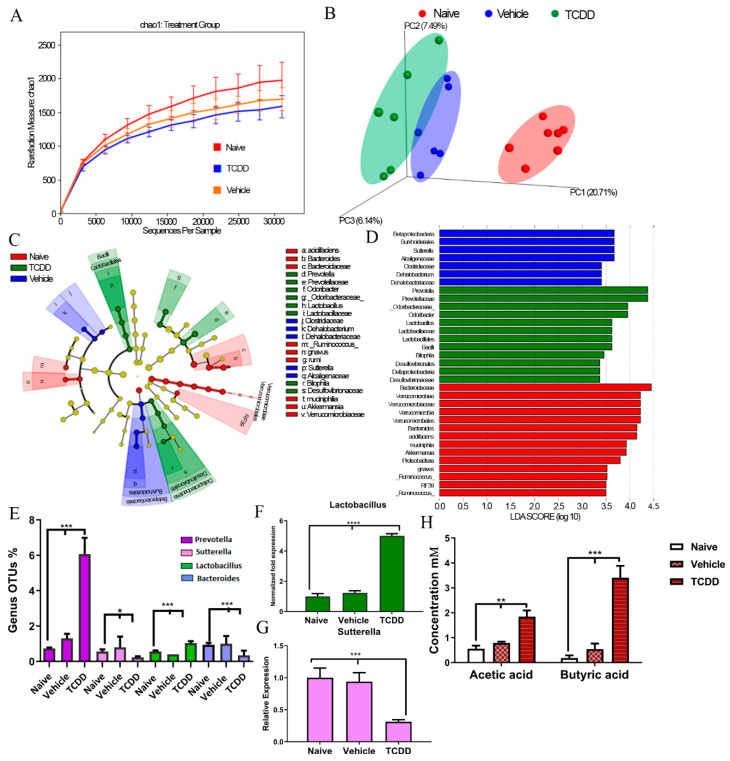
2,3,7,8-tetrachlorodibenzo-p-dioxin (TCDD) treatment alters microbiome composition. C57BL/6 mice were treated with TCDD (10 µg/kg) or the vehicle; 3 days later, feces were collected for 16S rRNA sequencing. (**A**) Rarefaction curves depicting alpha diversity within groups (Chao1 index) of naïve (*n* = 7), vehicle (*n* = 6), and TCDD treatment groups (*n* = 6) are shown. (**B**) Three-dimensional principal coordinate analysis (PCoA) based on the unweighted UniFrac distance of all samples for three groups: naïve, vehicle and TCDD. (**C**) Linear discrimination analysis of effect size (LeFSE)-generated cladogram for operational taxonomic units (OTUs) showing the phylum, class, order, family, genus, and species from the outer to inner swirl, respectively. Red indicates enrichment in taxa in samples from naïve group, blue from the vehicle group, and green from the TCDD group. (**D**) LeFSE-generated linear discrimination analysis (LDA) scores for differentially expressed taxa. The threshold LDA score was set to 3.5. (**E**) Percentage of OTUs of significantly altered bacteria at the genus level. (**F**,**G**) qPCR validation with primers for *Lactobacillus* (**F**) *Sutterella*, and bacteria. (**H**) Concentration of butyric acid and acetic acid produced by the microbiota in the fecal contents. Bar graphs consists of vertical bars representing mean ± standard error of the mean (SEM). One-way analysis of variance (ANOVA) with Tukey’s multiple comparisons test was used to determine significance; * *p* < 0.05; ** *p* < 0.01; *** *p* < 0.001; **** *p* < 0.0001. Data are representative of at least two independent experiments.

TCDD is a well characterized high affinity ligand for AhR; therefore, we used TCDD to investigate how AhR activation alters the gut microbiota. 16S rRNA sequencing with the Illumina MiSeq platform was performed on feces from the following groups: wild-type mice (naïve), mice treated with corn oil (vehicle), and mice given 10 µg/kg intraperitoneal (i.p.) injections of TCDD. Data collected from sequencing showed that vehicle or TCDD-treated mice had decreased alpha diversity when compared with naïve controls, which was assessed by Chao1 rarefaction measurement (Figure 1A). Beta diversity from principal coordinate analysis (PCoA) also showed that vehicle and TCDD-treated mice had gut microbial compositions dissimilar to naïve mice; however, all groups clustered in their own respective treatment niches, suggesting that TCDD-treated mice had a distinct composition when compared with vehicle mice (Figure 1B). However, sequencing data and operational taxonomic unit (OTU) classification from the phylum to genus level showed that TCDD treatment had a marked effect on gut microbiome composition (Figures S1–S5).

In order to differentiate the significantly altered bacteria among all the experimental groups, linear discrimination analysis of effect size (LeFSE) analysis was performed on the OTUs from phylum to genus. The results showed that there were several bacteria found to be distinctly expressed in the naïve, vehicle, and TCDD groups. These included the genera *Bacteroides*, *Sutterella*, *Prevotella*, and *Lactobacillus* (Figure 1C,D). Among the LeFSE-identified bacteria, significantly altered OTUs between vehicle and TCDD-treated mice included *Prevotella*, *Sutterella*, *Lactobacillus*, and *Bacteroides* (Figure 1E). Specifically, after TCDD exposure, there was a significant increase in abundance of several *Prevotella* and *Lactobacillus* at the genus level; however, *Sutterella* and *Bacteroides* were significantly decreased. To confirm the sequencing results, we quantified bacteria abundance from feces of experimental groups using bacteria-specific primers by PCR. These results confirmed that there was a significant increase (>4-fold change) in *Lactobacillus* in TCDD-treated groups when compared with naïve and vehicle groups (Figure 1F). In addition, PCR validation experiments confirmed that there was a significant reduction in *Sutterella* (~50%) in TCDD-exposed mice (Figure 1G). The phylogenetic sequencing and PCR validation data clearly demonstrated that TCDD exposure caused alterations in the microbiome, such as changing the abundance of *Lactobacillus* and *Sutterella* populations in the gut.

In addition to the phylogenetic data, bacterial metabolomic short chain fatty acid (SCFA) production was evaluated in the fecal samples from experimental mice exposed to TCDD. Of all the SCFAs studied, only two were found to be significantly altered after exposure to TCDD, which included acetic acid and butyric acid. Mice injected with TCDD showed significant increases in both acetic acid and butyric acid (Figure 1H) when compared with naïve control or vehicle-treated mice. Taken altogether, these results showed that TCDD exposure leads to not only changes in the microbial phylogeny in the gut, but also alters some SCFA production as well.

### 2.2. TCDD-Induced Fecal Microbiota When Transferred into Antibiotic-Treated Mice Can Trigger MDSCs and Tregs

**Figure 2:**
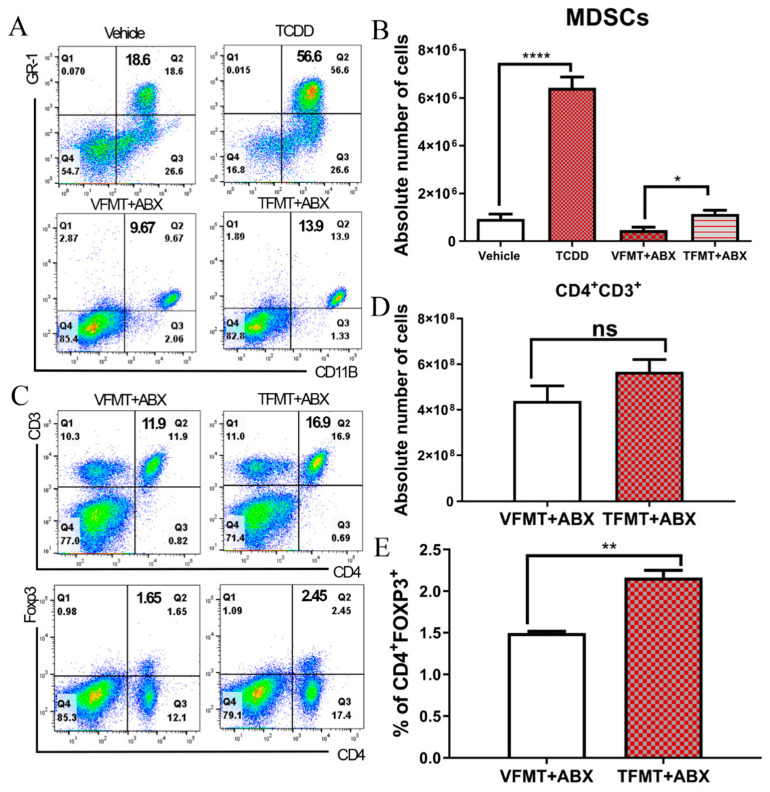
Effect of fecal microbiota transplantation (FMT) on myeloid derived-suppressor cells MDSCs and regulatory T-cells (Tregs). (**A**,**B**) Naïve C57BL6 mice received either the vehicle (*n* = 6) or TCDD (*n* = 6) and were analyzed for MDSCs (CD11b and Gr1) in the peritoneal cavity. Antibiotic-treated (ABX) mice (*n* = 6 per experimental group) received feces from vehicle-treated (FMT from vehicle mice (VFMT)+ABX) or TCDD-treated mice (FMT from TCDD mice (TFMT)+ABX), and peritoneal cavity cells were stained for MDSCs. (**A**) Representative flow plots showing the percentages of MDSCs. (**B**) Absolute number of MDSCs from the peritoneal cavity of experimental mice. (**C**–**E**) Cells from spleens of VFMT (*n* = 6) or TFMT (*n* = 6) recipient mice were processed to stain for T-cell markers. (**C**) Representative flow plot of T cells (CD4^+^ and CD3^+^; top panel) and Tregs (CD4^+^ and FoxP3^+^; bottom panel) in spleen cells of ABX recipient mice following FMT from vehicle (VFMT) or TCDD (TFMT) donors. (**D**) Total T cell number (CD4+and CD3+) in spleen cells of ABX recipient mice following FMT from vehicle (VFMT) or TCDD (TFMT) donors. (**E**) Percentage of Tregs from spleen cells of ABX recipient mice following FMT from vehicle (VFMT) or TCDD (TFMT) donors. Vertical bars represent mean  ±  SEM. One-way analysis of variance (ANOVA) with Tukey’s multiple comparisons test was used to determine significance for MDSC comparison with more than two groups; * *p* < 0.05; ** *p* < 0.01. For T-cells and Tregs with only two groups, significance was determined using Student’s *t* test; * *p* < 0.05; ** *p* < 0.01; **** *p* < 0.0001. Data are representative of at least two independent experiments.

TCDD exposure has been shown to regulate the immune response, particularly in suppressing the inflammatory T-cell-mediated response [14,22]. Recently, we found that activation of AhR by TCDD induces large numbers of CD11b^+^Gr-1^+^ MDSCs in the peritoneal cavity of mice [21], as also shown in the current report (Figure 2A upper panel and Figure 2B). To further understand the role of microbiota in TCDD-mediated MDSC induction, FMT experiments were performed in antibiotic-treated ABX-treated mice. After confirming that ABX treatment led to depletion of the gut microbiome (Figure S6), various treatments and FMT experiments were performed to evaluate the contribution of the microbial changes to MDSC induction. This included fecal microbiota transfer (FMT) from vehicle-treated mice (VFMT) into ABX mice (VFMT+ABX) or from TCDD-treated mice (TFMT) into ABX mice (TFMT+ABX). Interestingly, we observed that TFMT+ABX mice displayed higher proportions as well as increased numbers of MDSCs when compared with VFMT+ABX mice (Figure 2A lower panel and Figure 2B). The FMT experiments were repeated with consistent results (Figure S7). These results strongly suggested that the gut microbiome plays a role in TCDD-mediated induction of MDSCs. Moreover, when we injected TCDD directly into ABX mice, we were able to induce MDSCs (Figure S7), though basal levels of MDSCs were lower in ABX-treated mice compared with naïve mice (Figure 2A upper panel), thereby showing that the normal microbiota seen in naïve mice plays a role in MDSC induction by TCDD. We also injected butyrate into ABX mice to see if that would induce MDSCs and failed to detect any (Figure S7). The data that FMT from TCDD-treated mice into ABX mice could induce MDSCs and that TCDD could induce only a weak MDSC response in ABX mice, together demonstrated that induction of MDSCs by TCDD was dependent, at least in part, on the gut microbiota.

AhR activation by TCDD as well as MDSCs has been shown to induce Tregs [23,24]. To investigate if transplanted feces from TCDD-treated mice were able to induce Tregs, we harvested spleen cells from ABX-treated mice given TFMT or VFMT. We observed that TFMT mice did not exhibit any significant change in CD3^+^ and CD4^+^ cell numbers (Figure 2C upper panel and Figure 2D) but, interestingly, showed an increase in the numbers of CD4^+^ and FOXP3^+^ Tregs (Figure 2C lower panel and Figure 2D). These data showed that induction of Tregs in naïve mice by TCDD may also depend, at least in part, on the microbiota.

### 2.3. TCDD Reduces Cysteine Levels in Colon and Peritoneal Exudates

**Figure 3:**
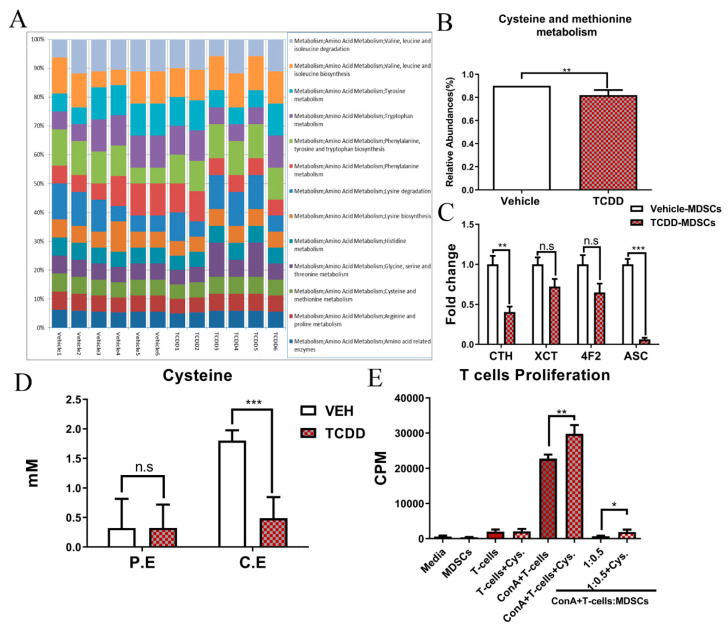
Role of cysteine in TCDD-mediated immunomodulation. C57BL/6 mice were treated with TCDD (10 µg/kg) or vehicle and feces were collected for 16S rRNA sequencing. (**A**) Phylogenetic Investigation of Communities by Reconstruction of Unobserved States (PICRUSt) data from Nephele depicting KEGG pathways altered in vehicle (*n* = 6) and TCDD (*n* = 6) gut microbiome. (**B**) Percent of OTUs attributed to cysteine and methionine metabolism. (**C**) qPCR quantification of alanine-serine-cysteine transporter (ASC) neutral amino acid transporter, XCT (light chain of antiporter Xc-), and 4F2 (heavy chain of antiporter Xc-) chains; CTH (cystathionase). MDSCs were selected by a phycoerythrin (PE) selection kit from the peritoneal fluid of vehicle or TCDD-treated groups. (**D**) Cysteine level quantification in peritoneal (P.E.) and colon exudate (C.E) in vehicle or TCDD mice. (**E**) Representative 3H-thymidine incorporation assay for T-cell proliferation in media deficient in cysteine or with cysteine. Student’s *t*-test (with only two groups) or one-way analysis of variance (ANOVA) with Tukey’s multiple comparisons test (comparing more than two groups) were used to compare between experimental groups; * *p* < 0.05; ** *p* < 0.01; *** *p* < 0.001. Data are representative of at least two independent experiments.

In addition to phylogenetic data obtained from Nephele of the 16s rRNA sequencing data, theoretical analysis of the gut microbiome’s metabolomic profile was determined using Phylogenetic Investigation of Communities by Reconstruction of Unobserved States (PICRUSt) (Figure 3A). Results from this in silico analysis assigning OTUs to Kyoto Encyclopedia of Genes and Genomes (KEGG) pathways showed that TCDD caused a significant reduction in cysteine and methionine metabolism (Figure 3B). To validate these findings, expression of cysteine-related genes was evaluated using PCR. The results showed that compared with vehicle-induced peritoneal MDSCs, TCDD-induced peritoneal MDSCs expressed less cystathionase (CTH), the enzyme responsible for converting intracellular methionine to cysteine (Figure 3C), as well as plasma membrane alanine-serine-cysteine transporter (ASC), neutral amino acid transporter Xc- and its light (XCT) and heavy chain (4F2) components, which are responsible for exporting cysteine and importing cystine from the environment (Figure 3C). Upon analysis of cysteine levels in the peritoneal and colon exudates of treated mice, it was found that cysteine levels were low in mice exposed to TCDD, while in the vehicle groups, colon exudates showed significantly higher levels (Figure 3D). In order to determine the role cysteine plays in MDSC function, proliferation assays were performed using ratios of MDSCs and T-cells in the presence or absence of cysteine. As shown, upon activation with ConA, adding cysteine in the media reduced the suppressive effect of MDSCs on the T-cells (Figure 3E). These data demonstrated that TCDD exposure significantly impacted cysteine metabolism and this, in turn, has effects on the ability of MDSCs to suppress the activated T-cell response.

### 2.4. TCDD-Mediated Effects on MDSCs and the Gut Microbiome Are Dependent on CXCR2

**Figure 4:**
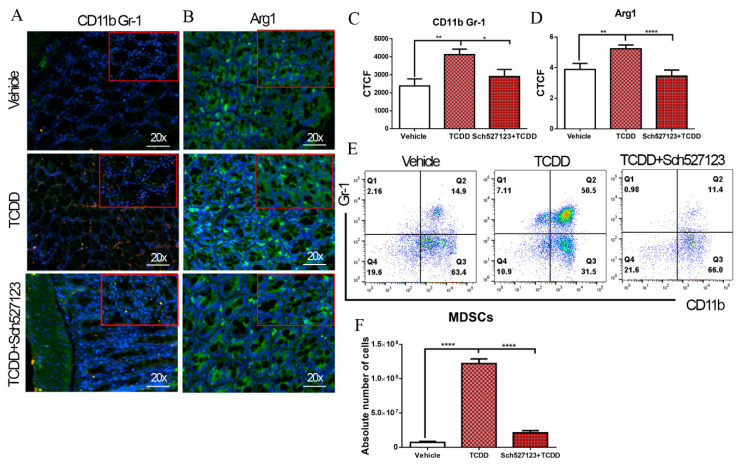
Role of CXC chemokine receptor 2 (CXCR2) in TCDD-mediated induction of MDSCs. Colon samples from three groups (vehicle, TCDD, and TCDD with the CXCR2 antagonist Sch5271230; *n* = 6 per experimental group) were fixed with 4% paraformaldehyde in PBS overnight and then sectioned to 5 µm thickness on coated slides. Slides were incubated with antibody detection of Gr-1, CD11b, and Arg1. (**A**) CD11b (green) and Gr-1 (red) fluorescence staining in the colon to detect MDSCs (green + red overlap = yellow). 20x, scale bar = 50 µm. (**B**) Arg1 fluorescence staining (green) to quantify Arg1 expression in the colon of three groups. 20x, scale bar = 50 µm (**C**,**D**) Statistical analysis of CD11b, Gr-1, and Arg1 expression in colon sections of three groups as measured in corrected total cell fluorescence (CTCF) using ImageJ software. (**E**) Representative flow plots of MDSCs in peritoneal fluid in vehicle, TCDD, and TCDD with the CXCR2 antagonist Sch527123 groups. (**F**) Absolute numbers of MDSCs in peritoneal fluid in vehicle, TCDD, and TCDD with the CXCR2 antagonist Sch527123 groups. One-way analysis of variance (ANOVA) and Tukey’s multiple comparisons test were used to compare among the groups. * *p* < 0.05; ** *p* < 0.01; **** *p* <0.0001. Data are representative of at least two independent experiments.

CXCR2 is a chemokine receptor that is important in the recruitment of MDSCs [25]. We recently published a report showing how TCDD-mediated MDSC suppression activity relates to CXCR2 [21]. Therefore, we investigated the effect of blocking CXCR2 on TCDD-mediated MDSC induction in the colon and peritoneal cavity. To test this notion, 50 mg/kg of the CXCR2 antagonist, Sch527123, was injected in mice one day before treatment with TCDD. Colon sections from experimental mice were taken 3 days after TCDD exposure and stained with MDSC-specific markers (CD11b, Gr-1 and Arg1). The results showed that CD11b, Gr-1, and Arg1 expression increased in the colon after injection with TCDD when compared with colons from vehicle-treated mice but this increase was lost in mice treated with the CXCR2 antagonist (Figure 4A–D). The results also showed that blocking CXCR2 prevented the accumulation of MDSCs in the peritoneal cavity after injection with TCDD (Figure 4E,F). Taken together with the FMT results, these data suggest that the TCDD-mediated effect on MSDCs is dependent on CXCR2.

## 3. Discussion

In the past several decades, numerous studies have shown that the exposure of laboratory animals to TCDD leads to profound immunosuppression [26,27,28,29]. More recent studies have shown that AhR activation by TCDD can suppress the immune system in mice by way of induction of Tregs [30,31]. Previous studies from our lab have also shown that TCDD was able to attenuate the clinical and inflammatory markers of colitis [14]. The gut microbiome consists of trillions of bacteria, which are sensitive to many endogenous and exogenous factors including diet, age, health condition, lifestyle, and environmental exposures [32]. A fundamental role of the microbiome in the induction, education, and function of the host immune system is therefore understandable. In a mutually reciprocal relationship, microbial colonization in the host gut affects the development of the immune system, and subtle changes in the immune system also have effects on the gut microbiome composition [33]. There are few studies on how AhR activation or exposure to TCDD can directly or indirectly cause changes in the gut microbiota, bile acids, and SCFA metabolism [34,35]. Previous studies have shown that TCDD, when given orally to mice, caused a shift in mouse gut commensals [36,37]. TCDD was even shown to play a role in influencing a shift favoring bacteria that expressed antimicrobial resistance genes (ARGs) [13].

In the current study, we observed significant alterations in the gut microbiome 3 days after exposure to TCDD. These alterations were characterized by reductions in certain bacteria, such as *Sutterella*, while significant increases in the abundance of other bacteria were observed, such as *Lactobacillus*. Some studies have reported that a decrease in *Sutterella* and an increase in *Lactobacillus* are related to immune tolerance. Tang et al. observed that a reduction in *Alcaligenaceae* and *Sutterella* levels in normal mice after feeding with purple sweet potato polysaccharides caused an increase in anti-inflammatory cytokines IL-2 and IL-6 [38]. Similarly, Pena et al. found significant reductions in pro-inflammatory IFN-γ and TNF-α in the spleen of probiotic-treated mice using a mixture of *Lactobacillus paracasei* and *Lactobacillus reuteri*, which resulted in lessening the severity of colitis in IL-10-deficient mice infected with *Helicobacter hepaticus* [39].

Alterations in the gut microbiome also lead to changes in the bacterial metabolome, such as the production of SCFAs. SCFAs, like acetic acid, butyric acid, and propionic acid, are metabolic end products of undigested complex carbohydrates for bacterial fermentation in the colon [40]. We observed that the levels of two SCFAs, butyric acid and acetic acid, were significantly higher in mice exposed to TCDD. Butyrate is known to exhibit tolerance-inducing activities such as the induction of Treg cells, as well as other anti-inflammatory activities, including increased production of IL-22 [41]. Several studies, both in vivo and in vitro, have demonstrated that SCFAs inhibit histone deacetylases (HDACs), which results, in many cases, in the inactivation of nuclear factor-κB (NF-κB) and downregulation of a number of pro-inflammatory cytokines, like tumor necrosis factor (TNF) [42,43]. In addition, increased SCFAs could enhance the differentiation of peripheral Treg populations through HDAC9 inhibition and, consequently, attenuation of colitis in mice [44]. Gallausiaux et al. also showed that butyrate produced by gut commensal bacteria influence the proportion and activation of anti-inflammatory regulatory T-cells (Tregs) [45]. Given this information, the increase in SCFAs like butyrate after TCDD exposure could explain some of the mechanisms which drive the immune suppression of this environmental pollutant, such as Treg induction. However, we found that direct administration of butyrate into ABX mice failed to induce MDSCs.

Mammalian cells, including immune cells, require the essential amino acid cysteine for protein synthesis and proliferation [46,47]. Cysteine is generated by cells through two distinct pathways. One involves reducing intracellular disulfide-bonded cystine, which is imported through the plasma membrane transporter Xc- to form cysteine, which is eventually exported through the plasma membrane ASC transporter. Another pathway involves converting intracellular methionine to cysteine if the cells express the cystathionase enzyme. T-cells do not express Xc- and ASC, and thus they depend on antigen-presenting cells (APCs) such as macrophages and dendritic cells to obtain cysteine [48,49,50,51,52]. MDSCs sequester cystine and do not export cysteine because they express only Xc-. Therefore, a large number of MDSCs in the microenvironment creates competition for cysteine between MDSCs and other immune cells, which can lead to a reduction in cysteine levels, causing the suppression of T-cell proliferation [53]. In the current report, TCDD reduced cysteine and methionine metabolism, as evidenced through examination by PICRUSt; these results were validated, showing cysteine levels in the peritoneal cavity and colon exudates were decreased after TCDD exposure. The reason behind cysteine reduction after TCDD exposure could be attributed to sequestering by MDSCs, or it could also be attributed to an increase in bacteria, such as *Lactobacillus*, which use cysteine as a sulfur source to grow [54]. It was thus interesting that when cysteine was provided in culture, it reversed the suppression of T-cell proliferation mediated by MDSCs.

CXCR2 was shown to play a critical role in the induction of MDSCs. CXCR2 has been shown to play a critical role in MDSC migration to endometrial lesions through interactions with CXCL1, -2, and -5 [55]. The fact that blocking CXCR2 reduced MDSCs significantly both in the colon and peritoneal cavity of TCDD-treated mice confirmed the important role played by CXCR2 in inducing MDSCs in the colon. In an earlier study, we noted that TCDD can induce CXCR2 in naïve mice because it expresses several dioxin response elements (DREs) on its promoter [21]. Thus, AhR activation by TCDD involving DREs on the CXCR2 gene promoter may help induce CXCR2, which, in turn, triggers MDSCs.

Based on the current study, we suggest that AhR activation is a double-edged sword. On one hand, AhR is critical to maintain intestinal immune system homeostasis. Thus, defects in AhR signaling have been shown to trigger colitis and intestinal bowel disease both in humans and in experimental animals [56]. Our studies suggest that this may result at least in part from induction of MDSCs. On the other hand, environmental contaminants such as TCDD, which act as potent AhR agonists and remain in the system for a very long time, are well characterized for their properties to suppressing the immune response and inducing cancer [57]. Thus, such chronic AhR activation leading to induction of MDSCs may suppress anti-tumor immunity and promote tumor development and progression. Further studies are necessary to directly test the role of MDSCs triggered through AhR activation by different types of agonists in acute and chronic disease models.

In summary, the current report provides evidence that TCDD causes a shift in the resident gut microbiome, particularly through increasing *Lactobacillus* and decreasing *Sutterella* abundance. FMT experiments confirmed that TCDD-mediated changes in the gut microbiome altered the immune system, specifically by increasing the MDSCs, which resulted in decreased cysteine levels. Such events may promote an immunosuppressive response, thus providing evidence that AhR activation by TCDD alters the microbiome in such a way that it influences the immune system of the host.

## 4. Materials and Methods

### 4.1. Animals

Female C57BL/6 adult mice were purchased from Jackson laboratory (Bar Harbor, MA, USA). All the animals were housed in the Animal Research Facility (ARF) located at the University of South Carolina (USC) under pathogen-free conditions. Mice were cared for in accordance with the NIH guideline for use of laboratory animals under protocols approved by the Institutional Animal Care and Use Committee (IACUC) at USC (Approval number: 2202-100876-081414; Approval Date: 08/05/2016).

### 4.2. Chemicals and Reagents

TCDD was a kind gift from Dr. Steve Safe (Institute of Biosciences & Technology, Texas A&M Health Sciences Center, College Station, TX, USA). Culture medium reagents (RPMI 1640), bacitracin, gentamycin, ciprofloxacin, neomycin, penicillin, metronidazole, ceftazidime, streptomycin, and vancomycin were from Sigma-Aldrich (St. Louis, MO, USA). HEPES, l-glutamine, FBS, and PBS were purchased from Invitrogen Life Technologies (Carlsbad, CA, USA). The following antibodies were purchased from Biolegend (San Diego, CA, USA) and used for surface markers or intra-cellular and/or intra-nuclear staining: Alexa Fluor 700-conjugated anti-CD11b, BV510-conjugated-GR-1, BV785-conjugated anti-CD4, PE-conjugated anti-CD3, and BV510-conjugated anti-FOXP3. FC Block and monoclonal mouse IgG anti-Arg1 were purchased from BD Biosciences (San Diego, CA, USA). Monoclonal rat IgG antibodies of CD11b and Gr-1 were purchased from Biolegend (San Diego, CA, USA). The Cytofix/CytopermTM Fixation/Permeabilization kit was purchased from BD Biosciences. The True-Nuclear™ Transcription Factor Buffer Set was purchased from BioLegend. EasySep™ PE Positive Selection Kits were purchased from Stem Cell Technologies (Vancouver, BC, Canada). The *N*-acetyl-cysteine (NAC) and the CXCR2 antagonist Sch527123 were purchased from Sigma-Aldrich (St. Louis, MO, USA). The Cysteine Assay Kit was purchased from Abcam (Cambridge, UK).

### 4.3. TCDD Exposure and 16S rRNA Amplicon Sequencing

Female C57BL/6 mice between 6 and 8 weeks were injected i.p. with TCDD (10 µg/kg) or the vehicle (corn oil), as described previously [58,59]. Feces were collected from individual TCDD- or vehicle-treated mice or from naïve mice, 3 days after treatment and kept at −80 °C for later use. 16S rRNA sequencing and analysis were performed as previously described [60]. The QIAamp DNA Stool Mini Kit (Qiagen, Germantown, MD, USA) was used for DNA isolation from fecal pellets (100 mg) of the three groups following the protocol of the company (Qiagen). Genomic DNA samples were quantified by using the Nanodrop system (Thermo Scientific, Waltham, MA, USA) and kept t −80 °C for further use. Amplification of the 16S rRNA V3–V4 hypervariable region was carried out using the 16S V3 314F forward (5′-TCGTCGGCAGCGTCAGATGTGTATAAGAGACAGCCTACGGGNGGC WGCAG-3′) and V4 805R reverse primers (5′-GTCTCGTGGGCTCGGAGATGTGTATAAGA GACAGGACTACHVGGGTATCTAATCC-3′). The Illumina overhang adapter sequences added to locus-specific sequences were: forward overhang: 5′ TCGTCGGCAGCGTCAGATGTGTATAAGAGACAG; reverse overhang: 5′ GTCTCGTGGGCTCGGAGATGTGTATAAGAGACAG. The PCR program used were 3 min at 95 °C, followed by 25 cycles of 30 s at 95 °C, 30 s at 55 °C, and 30 s at 72 °C, then a final extension at 72 °C for 5 min. Each reaction mixture (25 μL) contained 50 ng of genomic DNA, 0.5 μL of amplicon PCR forward primer (0.2 μM), 0.5 μL of amplicon PCR reverse primer (0.2 μM), and 12.5 μL of 2× KAPA Hifi Hot Start Ready Mix. AMPure XP beads were used for each reaction to purify the 16S V3 and V4 amplicon away from free primers and primer dimer species. Attachment of dual indices and Illumina sequencing adapters was performed using the Nextera XTIndex Kit including 5 μL of amplicon PCR product DNA, 5 μL of Illumina Nextera XT Index Primer 1 (N7xx), 5 μL of Nextera XT Index Primer 2 (S5xx), 25 μL of 2 × KAPA HiFi Hot Start Ready Mix, and 10 μL of PCR-grade water. Amplification was carried out under the following program: 3 min at 95 °C, followed by 8 cycles of 30 s at 95 °C, 30 s at 55 °C, and 30 s at 72 °C, then a final extension at 72 °C for 5 min. Constructed 16S metagenomic libraries were purified with AM Pure XP beads and quantified with Quant-iTPicoGreen. Libraries were quantified using a fluorometric quantification method that uses dsDNA binding dyes. DNA concentration was calculated in nM based on the size of DNA amplicons as determined by an Agilent Technologies 2100 Bioanalyzer trace. Libraries were normalized and pooled to 40 nM based on quantified values. Pooled samples were denatured and diluted to a final concentration of 8 pM with a 30% PhiX (Illumina) control. Samples were then loaded, and the results were provided by MiSeq Reporter software (MSR). The metagenomics workflow classified organisms from V3 and V4 amplicons using a database of 16S rRNA data. The classification was based on the Greengenes database (http://greengenes.lbl.gov/). The output of this workflow was a classification of reads at several taxonomic levels: kingdom, phylum, class, order, family, genus, and species. The online 16S analysis software from the National Institutes of Health (Nephele) was used to analyze the sequencing data collected on the Illumina MiSeq. The groups of related DNA sequences were assigned to operational taxonomic units (OTUs), and output files were analyzed to determine gut microbial composition. The Phylogenetic Investigation of Communities by Reconstruction of Unobserved States (PICRUSt) option during Nephele analysis was used to examine differences in Level 2 (L2) and Level 3 (L3) KEGG pathways using the collected 16S rRNA sequencing data. In order to differentiate significant alterations within the gut microbiome from experimental samples, linear discrimination analysis of effect size (LeFSE) was used as previously described [61].

### 4.4. Short Chain Fatty Acid Analysis

SCFAs were quantified as previously described by Mehrpouya-Bahrami et al. [62]. In brief, 100 mg cecal contents were acidified by metaphosphoric acid and allowed to sit on ice for 30 min. After centrifugation of acidified samples at 12,000× *g* for 15 min at 4 °C, supernatants were collected and filtered using MC filters at 12,000× *g* for 4 min at 4 °C. MTBE (400 µL) from Sigma (650560) was added to each sample after transferring samples into glass vials. The samples were then centrifuged down at 1300 rpm (or 393× *g*) for 5 min at RT and the top organic layer was transferred to a new vial. The standard mixtures with the internal standard were used to determine the response factors and linearity for each SCFA standard acid. A HP 5890 gas chromatograph configured with a flame-ionization detector (GC-FID) for analysis of volatile organic compounds was used to detect the concentration of propionic, n-butyric, isovaleric, valeric, isobutyric, caproic, and n-heptanoic acid in the samples.

### 4.5. Depletion of the Gut Microbiota

Depletion of the gut microbiome was achieved using a cocktail of antibiotics (ABX) consisting of the following: bacitracin, 1 mg/mL; gentamycin, 170 µg/mL; ciprofloxacin, 125 µg/mL; neomycin, 100 µg/mL; penicillin, 100 U/mL; metronidazole, 100 µg/mL; ceftazidime, 100 µg/mL; streptomycin, 50 µg/mL; and vancomycin, 50 µg/mL, as previously described [63,64,65] ABX treatment lasted for 24 days and was supplied in the drinking water. Feces for transfer experiments were collected from individual mice under sterile conditions. To validate microbiome reduction, DNA was isolated using the QIAamp DNA Stool Mini Kit (Qiagen) and analyzed by performing agarose gel electrophoresis. DNA fragments were visualized by imaging Chemi-Blots on the Bio-Rad ChemiDoc XRS HQ. The band density of DNA from ABX-treated mice was compared with the band density of DNA from WT control mice. In addition, swabs from ABX-treated mice and control feces were cultured in aerobic and anaerobic conditions for 2 days. ABX treatment was stopped on Day 25, and the mice to be inoculated with fecal material were co-housed with vehicle and TCDD donors on Day 26, followed by all subsequent treatments given to the mice on Day 27.

### 4.6. Fecal Material Transplantation

FMTs were performed to determine the effects of TCDD on the gut microbiome after TCDD treatment. Donor mice were divided into two groups (*n* = 8 per group). One group was injected with 10 µg/kg i.p. injections of TCDD (T), and the second group was treated with corn oil as the vehicle (V). After 3 days, colon contents were collected under sterile and anaerobic conditions for dilution in sterile PBS. ABX-treated mice were divided into 5 groups (*n* = 4–5 per group) and treated as follows: untreated, treated with butyrate in their drinking water (1% sodium butyrate), treated with TCDD (10 µg/kg) (TCDD), treated with FMTs from vehicle-treated mice (VFMT), and treated with FMT from TCDD-treated mice (TFMT). The mice were euthanized after 3 days using isoflurane overdose to collect cells from peritoneal exudates, spleens, and blood.

### 4.7. Flow Cytometry to Evaluate Immune Cell Phenotypes

MDSCs and MDSC subsets were stained and identified as described by us previously [66]. Cells were harvested from the peritoneal cavity of recipient and treated mice and stained with fluorescent-labeled antibodies (Biolegend and BD Biosciences) for phenotyping. Antibodies included Alexa Fluor 700-conjugated anti-CD11b and BV510-conjugated-Gr-1 (San Diego, CA, USA) to determine MDSCs (CD11b^+^Gr-1^+^). For T helper and transcription factor FOXP3 staining, the antibodies used were BV785-conjugated anti-CD4, PE-conjugated anti-CD3, and BV510-conjugated anti-FOXP3 to detect Tregs (CD4^+^FoxP3^+^). Flow cytometry analysis was performed using BD FACs Celeste and FlowJo software from ThermoFisher Scientific.

### 4.8. Real-Time PCR

DNA was isolated from the feces of experimental groups using QIAamp DNA Stool kit from Qiagen (Valencia, CA, USA), and samples were diluted to 1 ng/µL concentrations. The MiScript primer assays kit and the miScript SYBR Green PCR kit from Qiagen were used to perform PCRs following the protocols provided by the company. *Sutterella* and *Lactobacillus* PCR primers were purchased from IDT Technologies with primer sequences based on previous publications [67,68]. Cystathionase, XCT, 4F2, and ASC primers were designed based on a previous publication [53]. The PCR products, generated from mouse gene-specific primer pairs or bacteria-specific primers pairs, were visualized with UV light-performing electrophoresis (1.2% agarose gel). The band intensity of PCR products was determined using the ChemiDoc image analysis system from Bio-Rad (Bio-Rad, Hercules, CA, USA). The expression of the above genes were normalized against PCR products generated from the mouse housekeeping gene GAPDH or against PCR products generated from the Eubacteria gene (internal controls) as previously reported [69].

### 4.9. Purification of MDSCs

TCDD-induced MDSCs from the exudates of peritoneal cavities were purified as previously described using the selection of Gr-1^+^ MDSCs [70]. In brief, peritoneal exudates were collected from TCDD-exposed mice; after washing the cells 2 times with PBS, cells were labeled with a PE-conjugated Gr-1 antibody from Biolegend and magnetically sorted using a Positive Mouse PE Selection kit from Stem Cell Technologies (Cambridge, MA, USA) following the instructions from the manufacturer.

### 4.10. [3H] Thymidine Incorporation Assay

To measure the proliferation of T-cells, splenocytes (5 × 10^5^) from C57BL/6 naïve mice were cultured in the presence of Con A (2 µg/mL) in media deficient in *N*-acetyl-cysteine (NAC) or with NAC (0.5 Mm) in a 96-well round-bottom plate. The cells were cultured alone or cultured together with TCDD-induced peritoneal MDSCs at the ratio of 1:0.5 overnight. [3H] thymidine (1 µCi/well) was added to the cell cultures, and after 18 h, radioactivity was measured using a MicroBeta Trilux liquid-scintillation counter (PerkinElmer Life and Analytical Sciences, Waltham, MA, USA).

### 4.11. Detection of Cysteine Levels in Peritoneal Exudate and Colon Exudate

Cysteine concentration was assessed in peritoneal and colon exudates collected from mice that received TCDD treatment using a fluorometric Cysteine Assay Kit (ab211099) from Abcam following the protocol from the manufacturer. Delta corresponding fluorescence values (∆RFU) were calculated and applied to the cysteine standard curve to calculate the reaction concentration.

### 4.12. Fluorescence Staining of Colon Tissue Sections

Colon tissue samples from three groups (vehicle, TCDD, and TCDD+Sch527123) were fixed in 4% paraformaldehyde diluted in PBS overnight. Fixed colons were sectioned (5 µm thick) and placed on coated slides. Slides were incubated for 30 min in glycine 0.1% and 1× Triton for tissue permeabilization for Arg1 staining or with glycine 0.1% only for CD11b and Gr-1 staining. Slides were then incubated with primary mouse anti-Arg1 antibodies or primary rat anti-CD11b and anti-Gr-1 antibodies purchased from Cell Signaling (Danvers, MA, USA) at 4 °C overnight, followed by a 2 h incubation at room temperature with secondary Alexa Fluor 488 goat anti-mouse IgG antibody for Arg1, Alexa Fluor 488 goat anti-rat IgG for CD11b, and Cy5 goat anti-rat IgG antibody for Gr-1. Fluorescent imaging of colon sections was taken using a Leica DM 2500 optical microscope from Leica Microsystems (Buffalo Grove, IL, USA). The quantification of cell markers was calculated as corrected total cell fluorescence (CTCF) using Image J software (National Institutes of Health and the Laboratory for Optical and Computational Instrumentation).

### 4.13. Statistical Analysis

GraphPad Prism software version 6.01 (San Diego, CA, USA) was used for statistical analysis. Student’s *t*-test was used for paired observations if the data followed a normal distribution to compare between two groups, while one-way analysis of variance (ANOVA) was used to compare among more than two groups. A *p*-value of ≤0.05 was considered statistically significant. For all experimental results, data were collected from at least two independent experiments with consistent results unless otherwise stated.

## Supplementary Materials

Supplementary Materials can be found at https://www.mdpi.com/1422-0067/21/24/9613/s1.

## Abbreviations

TCDD	2,3,7,8-Tetrachlorodibenzo-p-dioxin
AhR	Aryl hydrocarbon receptor
MDSCs	Myeloid-derived suppressor cells
ABX	Antibiotic treatment
FMT	Fecal microbiota transfer
VFMT	Vehicle fecal microbiota transfer
TFMT	TCDD fecal microbiota transfer
CXCR2	C-X-C motif chemokine receptor 2
SCFA	Short-chain fatty acid
NAC	*N*-acetyl-cysteine
Arg1	Arginase 1
CTH	Cystathionase
ASC	Alanine-serine-cysteine transporter
FOXP3	Forkhead box P3
Tregs	Regulatory T-cells
PICRUST	Phylogenetic investigation of communities by reconstruction of unobserved states
OTU	Operational taxonomic unit
