# Supplementary material for: AhR Activation Leads to Alterations in the Gut Microbiome with Consequent Effect on Induction of Myeloid Derived Suppressor Cells in a CXCR2-Dependent Manner
Source: Int J Mol Sci. 2020 Dec 17;21(24):9613. doi: 10.3390/ijms21249613 (PMC7767008; doi:10.3390/ijms21249613)
Supplement: Supplementary file 1 [file ijms-21-09613-s001.pdf]

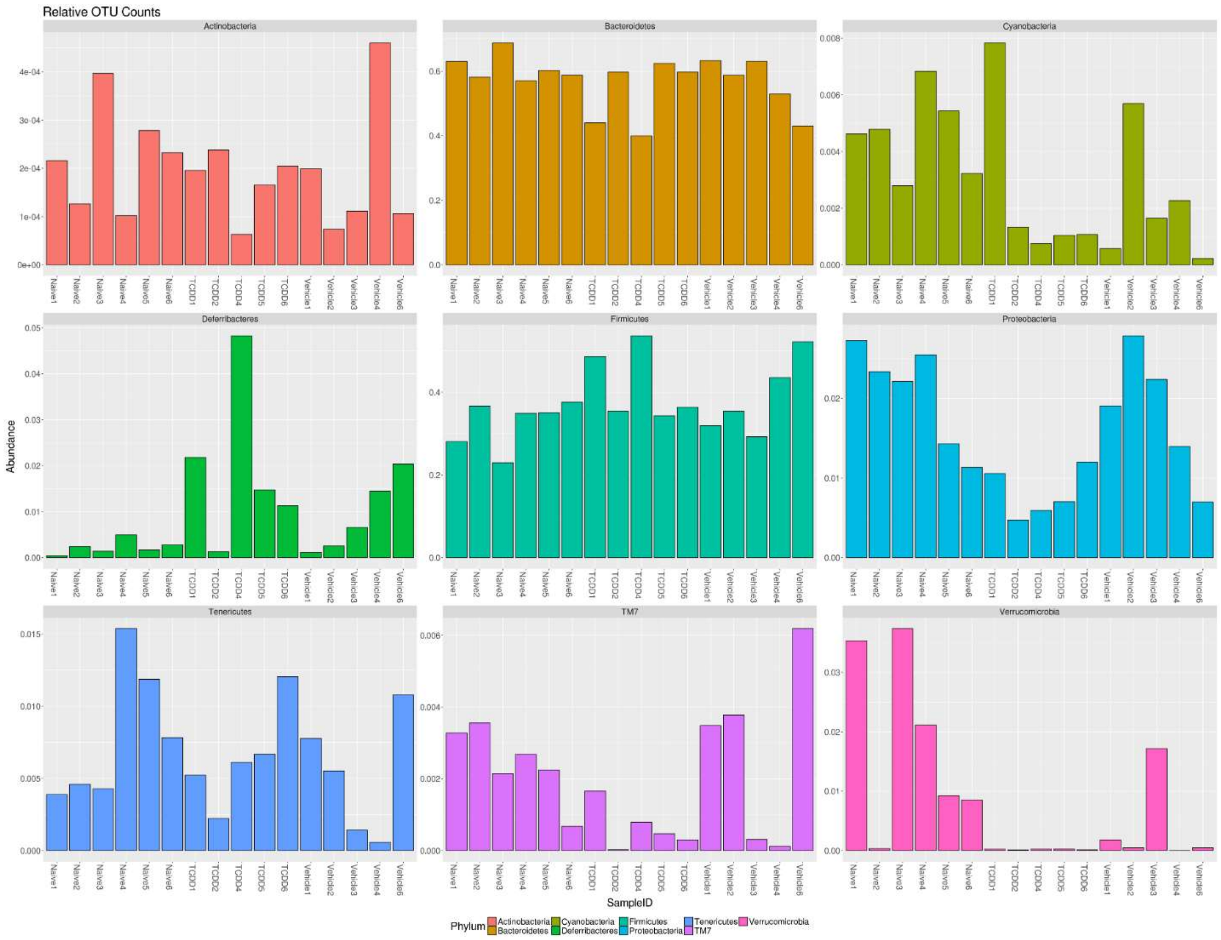

Supplementary Figure 1. *Relative OTU abundances at the Phylum level.* Mini blot bar from Nephele analysis showing 16s rRNA sequencing data depicting relative OTU abundance at the phylum level. Sequenced reads from cecal flushes of Naive (n=6), Vehicle (n=5), and TCDD (n=5) were uploaded into the Nephele platform for 16S OTU analysis. Individual sample bar graphs and figure legends were generated in the Nephele output files.

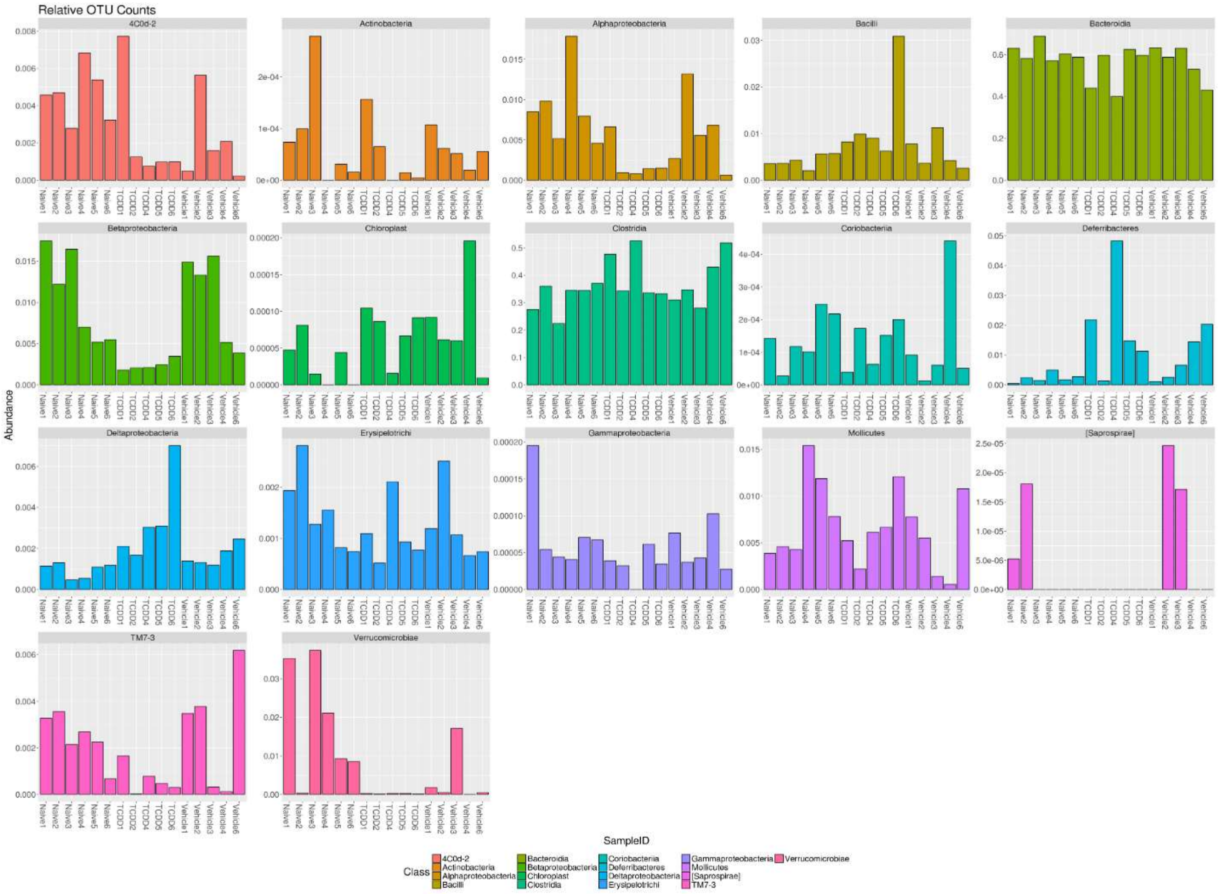

Supplementary Figure 2. *Relative OTU abundances at the Class level.* Mini blot bar from Nephele analysis showing 16s rRNA sequencing data depicting relative OTU abundance at the class level. Sequenced reads from fecal content of Naive (n=6), Vehicle (n=5), and TCDD (n=5) were uploaded into the Nephele platform for 16S OTU analysis. Individual sample bar graphs and figure legends were generated in the Nephele output files.

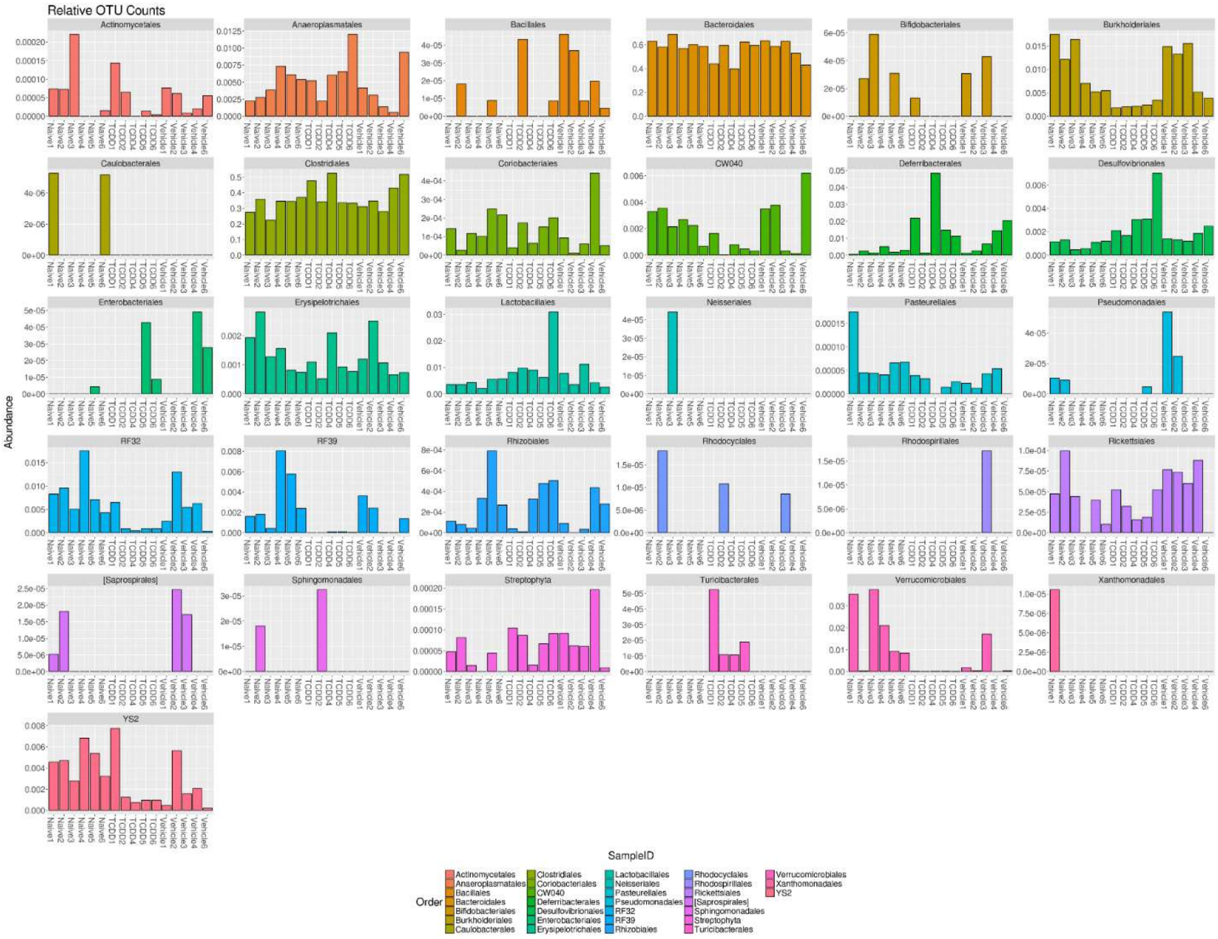

Supplementary Figure 3. *Relative OTU abundances at the Order level.* Mini blot bar from Nephele analysis showing 16s rRNA sequencing data depicting relative OTU abundance at the order level. Sequenced reads from fecal content of Naive (n=6), Vehicle (n=5), and TCDD (n=5) were uploaded into the Nephele platform for 16S OTU analysis. Individual sample bar graphs and figure legends were generated in the Nephele output files.

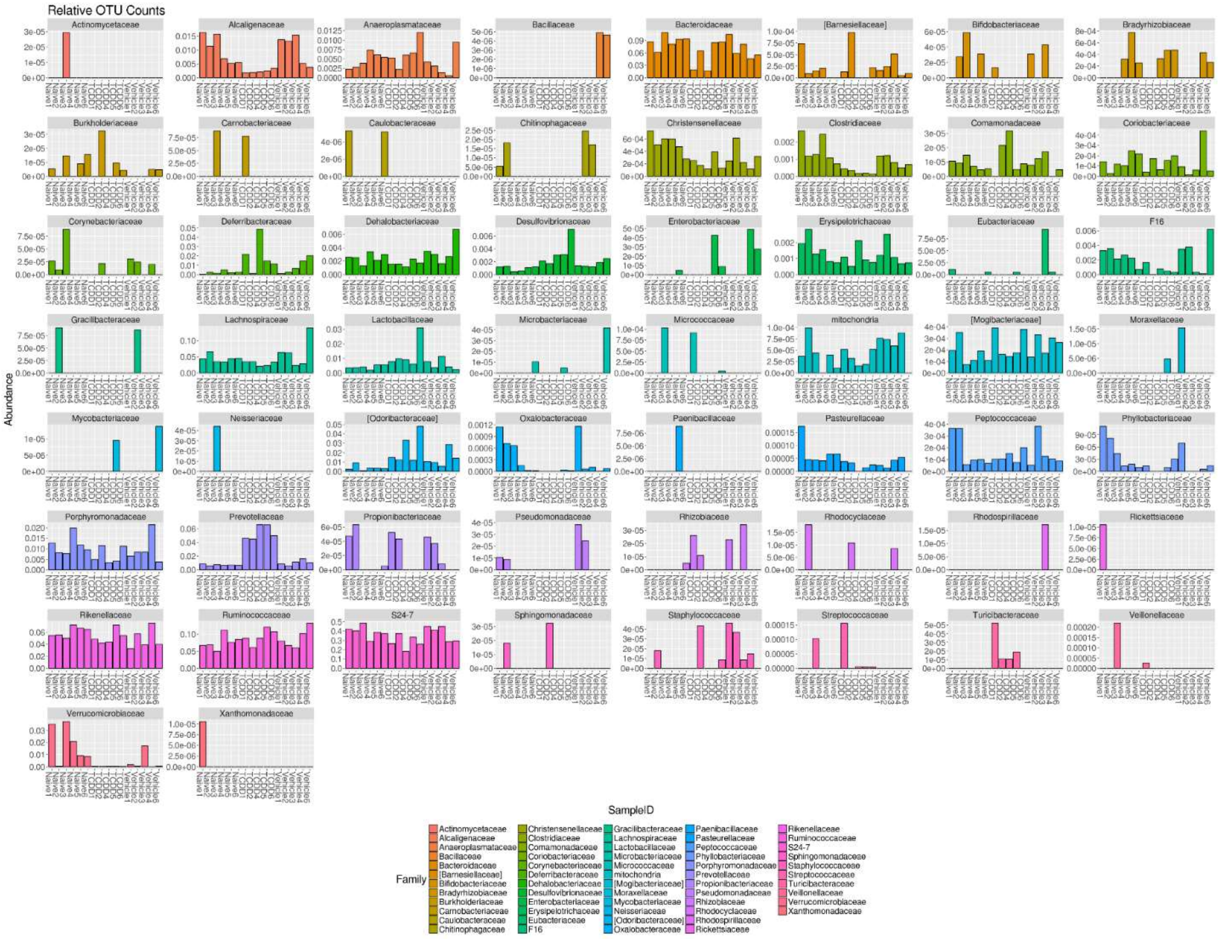

Supplementary Figure 4. *Relative OTU abundances at the Family level***.** Mini blot bar from Nephele analysis showing 16s rRNA sequencing data depicting relative OTU abundance at the family level. Sequenced reads from fecal content of Naive (n=6), Vehicle (n=5), and TCDD (n=5) were uploaded into the Nephele platform for 16S OTU analysis. Individual sample bar graphs and figure legends were generated in the Nephele output files.

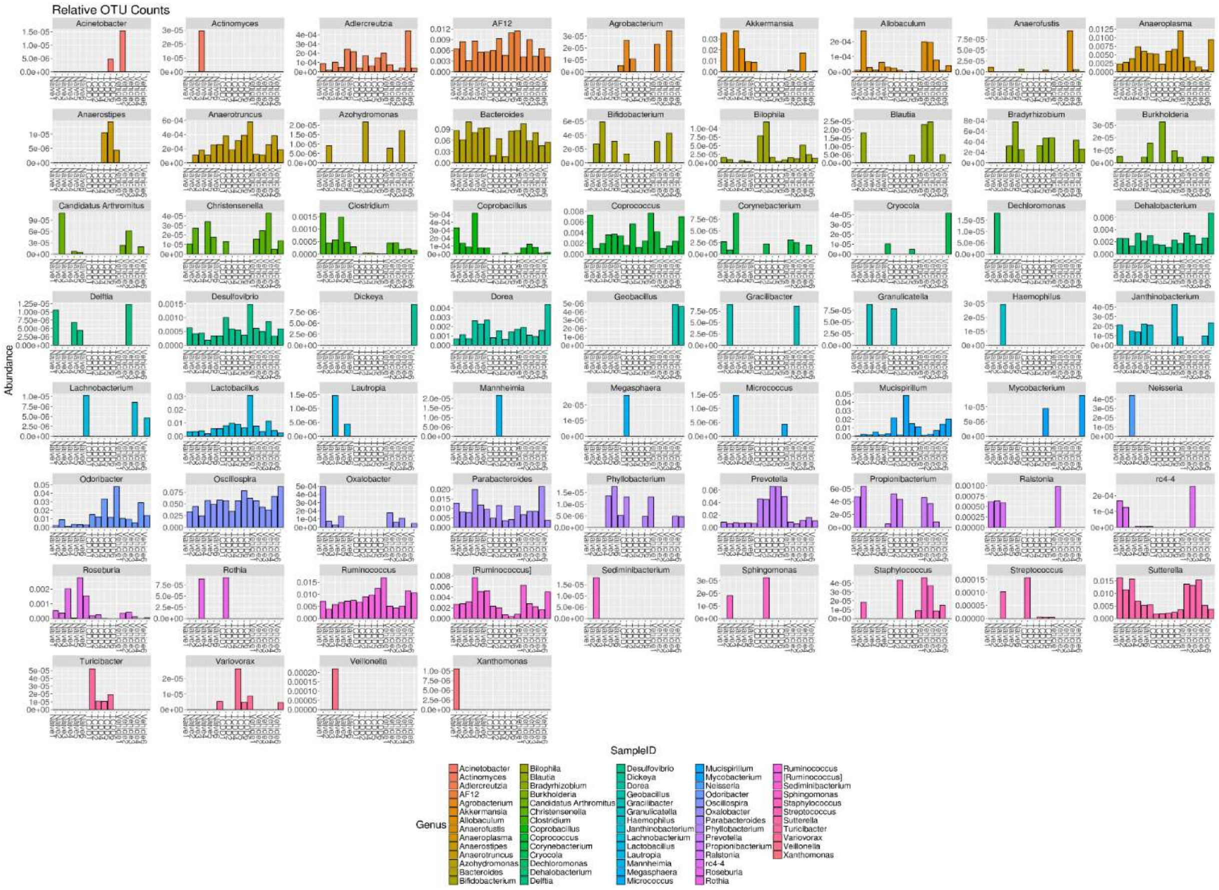

Supplementary Figure 5. *Relative OTU abundances at the Genus level***.** Mini blot bar from Nephele analysis showing 16s rRNA sequencing data depicting relative OTU abundance at the genus level. Sequenced reads from fecal content of Naive (n=6), Vehicle (n=5), and TCDD (n=5) were uploaded into the Nephele platform for 16S OTU analysis. Individual sample bar graphs and figure legends were generated in the Nephele output files.

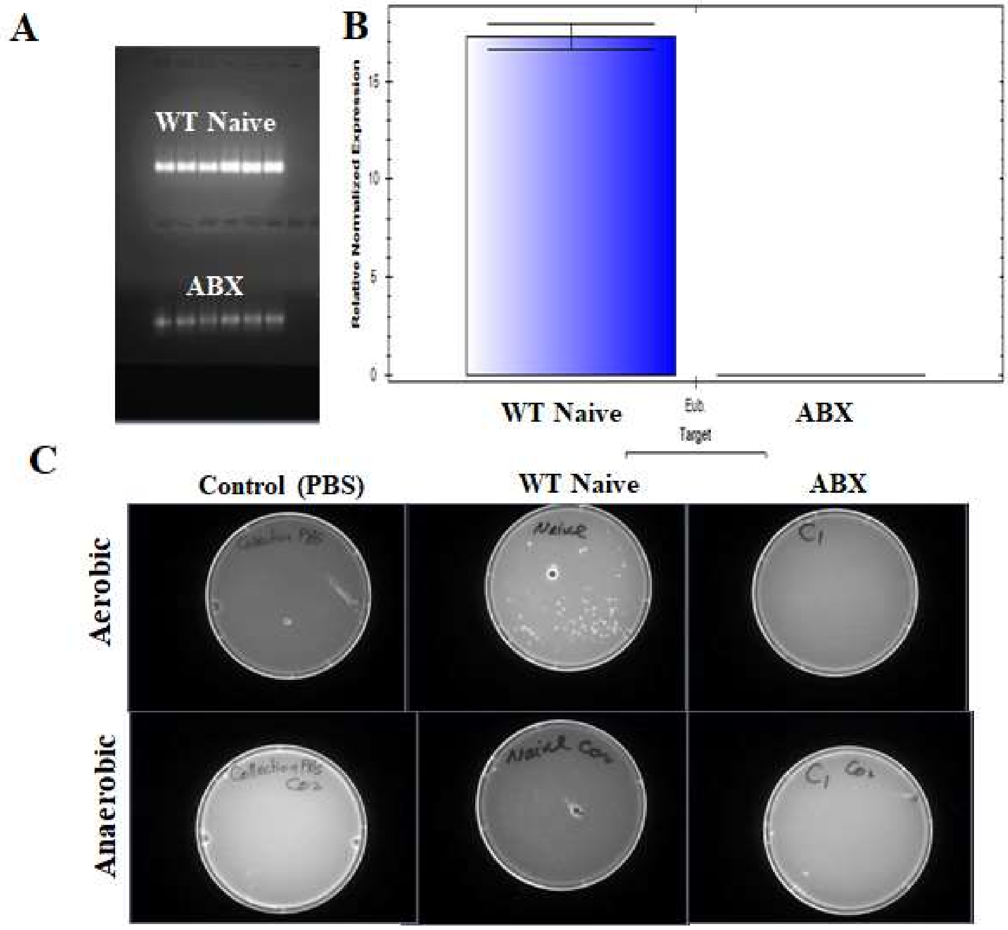

Supplementary Figure 6. *Confirmation of microbiota depletion after ABX treatment***.** Naïve mice were treated with a cocktail of antibiotics for 3 weeks prior to FMT experiments. (A) UV agarose gel electrophoresis image of genomic DNA in naïve WT (n=6) and ABX mice (n=6) showing PCR expression of Eubacteria. (B) Relative normalized expression of Eubacteria in WT Naïve or ABX mice using qRT-PCR (C) UV images of culture plates with swabs from fecal samples from control (n=6), WT Naïve, and ABX mice in aerobic (top) and anaerobic conditions (bottom).

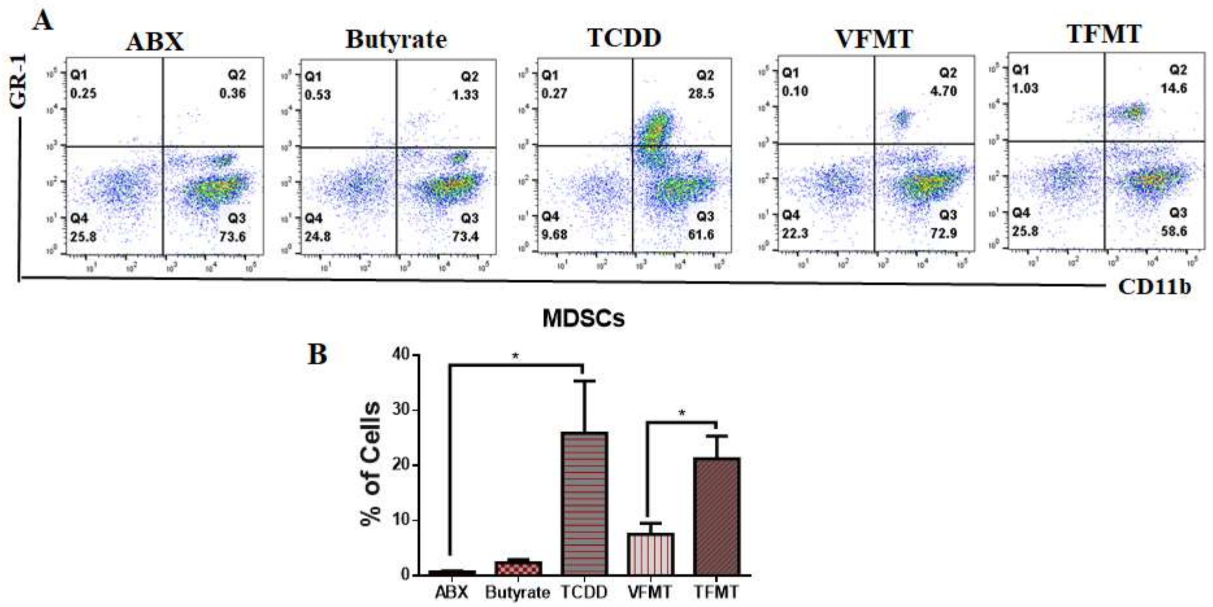

Figure S7. *Effect of TCDD or FMT on MDSC induction in ABX mice*. These experiments were carried out as described in Fig 2 legend. ABX (n=6 experimental per group) mice were administered TCDD alone, sodium butyrate alone, FMT from vehicle-treated (VFMT) or TCDD-treated (TFMT) donor mice. (A) Peritoneal cavity cells were stained for CD11b Gr-1 for MDSCs. (B) Total percentage of MDSCs in the ABX-treated mice given Butyrate, TCDD, and FMTs. Vertical bars show mean ± SEM percentage of cells. One-way analysis of variance (ANOVA) with Tukey's multiple comparisons test was used to determine significance; $^{*}p < 0.05$; $^{**}p < 0.01$; $^{***}p < 0.001$.
